# Collaborative Action of Toll-Like and Nod-Like Receptors as Modulators of the Inflammatory Response to Pathogenic Bacteria

**DOI:** 10.1155/2014/432785

**Published:** 2014-12-01

**Authors:** Javier Oviedo-Boyso, Alejandro Bravo-Patiño, Víctor M. Baizabal-Aguirre

**Affiliations:** Molecular Immunology and Signal Transduction Laboratory, Centro Multidisciplinario de Estudios en Biotecnología, Facultad de Medicina Veterinaria y Zootecnia, Universidad Michoacana de San Nicolás de Hidalgo, Km. 9.5 s/n Carretera Morelia-Zinapécuaro, La Palma, Tarímbaro, C.P. 58893 Morelia, MICH, Mexico

## Abstract

Early sensing of pathogenic bacteria by the host immune system is important to develop effective mechanisms to kill the invader. Microbial recognition, activation of signaling pathways, and effector mechanisms are sequential events that must be highly controlled to successfully eliminate the pathogen. Host recognizes pathogens through pattern-recognition receptors (PRRs) that sense pathogen-associated molecular patterns (PAMPs). Some of these PRRs include Toll-like receptors (TLRs), nucleotide-binding oligomerization domain-like receptors (NLRs), retinoic acid-inducible gene-I- (RIG-I-) like receptors (RLRs), and C-type lectin receptors (CLRs). TLRs and NLRs are PRRs that play a key role in recognition of extracellular and intracellular bacteria and control the inflammatory response. The activation of TLRs and NLRs by their respective ligands activates downstream signaling pathways that converge on activation of transcription factors, such as nuclear factor-*kappa*B (NF-*κ*B), activator protein-1 (AP-1) or interferon regulatory factors (IRFs), leading to expression of inflammatory cytokines and antimicrobial molecules. The goal of this review is to discuss how the TLRs and NRLs signaling pathways collaborate in a cooperative or synergistic manner to counteract the infectious agents. A deep knowledge of the biochemical events initiated by each of these receptors will undoubtedly have a high impact in the design of more effective strategies to control inflammation.

## 1. Introduction

All living organisms are constantly challenged by microorganisms and a variety of particles (from air pollution and cellular stresses) that represent a health threat. To counteract this burden, the innate immune system needs to react promptly and adequately to eliminate them, while at the same time to preserve tissue normal function. In the last decade there has been an enormous progress in the study of the molecular mechanisms that allow the host to fight against any antigenic stimuli and to keep internal homeostasis. In general, the innate immune host defense includes three essential sequentially events: (1) microbial recognition, (2) activation of signaling pathways, and (3) effector mechanisms.

Hosts are able to recognize distinct PAMPs present in microorganisms through a wide variety of PRRs. To date, a broad range of PRRs have been reported that include TLRs, NLRs, RLRs, and CLRs (for a complete description, see [1]). The different subcellular localization of PRRs and the broad array of PAMPs that can be recognized by them, allows the host to sense a large number of pathogen bacteria and develop an adequate immune response. Upon recognition of PAMPs by PRRs signal transduction pathways are activated that converge on transcription factors, such as NF-*κ*B, AP-1 or IRFs. Activation of these transcription factors regulates the inflammatory and innate immune response through the expression of proinflammatory mediators and antimicrobial effectors. Since some pathogenic bacteria possess PAMPs that can be simultaneously recognized by several PRRs and this leads to the activation of common transcription factors, it is likely that a collaborative response among different signaling molecules may exert regulatory functions after recognition of pathogenic bacteria. Therefore, the aim of this review is to discuss recent findings on the collaborative activity of TLRs and NLRs in the modulation of the inflammatory response induced by virulence factors of pathogenic bacteria.

## 2. Recognition of Pathogenic Bacteria by the Host 

Animals, including humans, respond to a wide range of antigenic stimuli in order to preserve their homeostatic conditions [2]. Professional (macrophages, neutrophils, and dendritic cells) and nonprofessional (epithelial and endothelial cells) phagocytes express various PRRs that recognize PAMPs as well as other nonbiological stimuli [3, 4]. Among the most important PAMPs are lipoteichoic acid (LTA) and peptidoglycan (PGN) from Gram-positive bacteria, lipopolysaccharide (LPS) from Gram-negative bacteria, lipoarabinomannan (LAM), lipopeptides, lipoglycans and lipomannans from mycobacteria, glycosylphosphatidylinositol (GPI), anchored lipids from* Trypanosoma cruzi*, zymosan isolated from yeast, profilin from* Toxoplasma gondii*, and DNA from bacteria and mycobacteria [5–8]. To date, a broad range of PRRs have been reported, such as TLRs, NLRs, RLRs, and CLRs.

## 3. Toll-Like Receptors

TLRs constitute a family of receptors, with different specificities, 10 in humans and 12 in murines. They are able to recognize a structural diversity of PAMPs like glycans, lipids, proteins, lipoproteins, and nucleic acids [9] and are widely known as key players of the inflammatory and innate immune response [10]. TLRs are expressed in various cellular compartments. TLR1, TLR2, TLR4, TLR5, TLR6, and TLR11 are predominantly expressed on the cell surface, although it has been reported that after ligand binding, TLR2 and TLR4 are internalized to phagosomes. TLR3, TLR7, TLR8, TLR9, and TLR13 are expressed in intracellular vesicles such as the endoplasmic reticulum, endosomes, lysosomes, and endolysosomes and mainly recognize nucleic acid [11, 12]. TLRs are expressed in a broad variety of cells such as dendritic cells, macrophages, neutrophils, monocytes, T and B cells, epithelial cells, endothelial cells, fibroblasts, and even neural cells (Table 1) [13–18]; however, each type of cell contains a specific set of TLRs [19–22].

Monocytes, mature macrophages, and dendritic cells virtually express all TLRs, while TLR4 and TLR5 are also expressed in intestinal epithelium. Moreover, it has been reported that plasmacytoid dendritic cells and mast cells express TLR7 and TLR8, respectively [19, 23–25]. Interestingly, TLR12 colocalize with TLR11 in endoplasmic reticulum of macrophages [26, 27]. TLR signaling has been shown to be involved in several functions in gut, such as epithelial cells proliferation, IgA production, maintenance of tight junction, antimicrobial peptide expression and pathogen bacteria recognition [20, 28–32]. Airway epithelial cells express TLR1, TLR2, TLR3, TLR4, TLR5, and TLR6 [33–35], although TLR4 has a constitutive expression and intracellular localization [36]. However, TLR7 and TLR9 are only expressed in primary airway epithelial cells [21, 37].

TLRs also play a major role in cutaneous host defense against microorganisms [22, 38]. Normal keratinocytes of the epidermis constitutively express TLR1, TLR2, and TLR5, while TLR3 and TLR4 are barely detectable [38, 39]. However, other work showed evidence that keratinocytes express TLR4 at the mRNA and protein level [40, 41]. Latter it has been shown that keratinocytes respond to double-strand RNA (dsRNA) and express a functional TLR3 [42, 43]. Interestingly, TLR6 and TLR9, but not TLR7 and TLR8 [44], are also expressed by keratinocytes [45].

## 4. NOD1 and NOD2

NLRs constitute a family of intracellular receptors that detects PAMPs and endogenous molecules. This family contains 16 members that have been categorized into five structurally different subfamilies: NLRA, with an acidic transactivation domain; NLRB, with a baculovirus inhibitor of apoptosis protein repeat; NLRC, with a CARD domain; NLRP, with a Pyrin domain; and NLRX that contains an uncharacterized domain [46]. Probably, the NLRC receptors NOD1 and NOD2 that recognize intracellular bacterial products, as well as NLRPs that respond to multiple stimuli to form a multiprotein complex termed the NALP-inflammasome, are the best characterized so far [47–49] and will be discussed below.

The expression of NLRs has been described in a variety of cellular types (Table 1), for example, NOD1 is ubiquitously expressed in various cell types such as macrophages, human mononuclear cells, intestinal epithelial cells, and dendritic cells, while NOD2 is expressed at higher levels in phagocytic cells and Paneth cells of the small intestine [50–52]. NOD1 and NOD2 have emerged as key pathogen recognition molecules of the innate immune responses [53, 54]. Since the first report of NOD1 as a receptor of invasive* Shigella flexneri *[55, 56], other works have shown that NOD1 is the receptor involved in the cytosolic recognition of invasive Gram-negative bacteria or PGN delivered into the epithelial cells through outer membrane vesicles (OMV) derived from these types of bacteria or injected through type three secretion systems (TTSS) [57–60]. The participation of NOD1 in Gram-negative bacteria sensing might represent a selective advantage to the host because these types of bacteria are a common threat of the epithelial cells lining the intestinal mucosa [61]. NOD1 and NOD2 have been shown to detect enteric bacteria such as* Shigella*,* Salmonella*,* Listeria*,* Yersinia*, pathogenic* Escherichia coli* strains, and* Mycobacterium* species [62]. The expression of NOD2 has also been associated with the chronic intestinal inflammation in Crohn's disease, where stimulation with muramyl dipeptide (MDP) seems to play an important role [63].

In contrast to NOD1 that is expressed in a wide range of cells and tissues, the expression of NOD2 seems to be restricted to macrophages, neutrophils, dendritic cells, and lung epithelium [64, 65]. Specifically in the lung, several reports have shown that NOD1 is expressed in epithelial cells, endothelial cells, human airway smooth muscle cells, and leukocytes [66–69] and responds to pathogens such as* Chlamydophila pneumoniae*,* Legionella pneumophila*,* Klebsiella pneumoniae*,* Haemophilus influenzae*, and* Pseudomonas aeruginosa* [58, 70–73]. NOD2 has been found mainly in macrophages, neutrophils, and bronchial cells [70, 74–76] and senses* Streptococcus pneumoniae*,* Staphylococcus aureus*,* E. coli*,* C. pneumoniae*, and* M. tuberculosis* [77–79].

## 5. NLR Inflammasomes

NLRPs are a subgroup of NLRs constituted by proteins such as NLRP1, NLRP3, NLRP4, NLRP6, NLRP7, and NLRP12 that are involved in the formation of multiprotein complexes termed inflammasomes [80]. These complexes consist of one or two NLR proteins, the adapter molecule apoptosis associated speck-like containing a CARD domain (ASC) and pro-caspase-1 [81]. These inflammasomes might sense several microbial products and a variety of stress and damage associated endogenous signals. Probably the best characterized inflammasome is the one formed by the NLRP3 scaffold, the ASC adaptor and caspase-1 [82], and its expression is induced by inflammatory cytokines and TLR agonists in myeloid cells and human bronchial epithelial cells [82]. As the other inflammasomes, the NLRP3 inflammasome mediates the caspase-1-dependent conversion of pro-IL-1*β* and pro-IL-18 to IL-1*β* and IL-18 and are involved in a form of cell death termed pyroptosis [83].

NLRPs respond to a broad variety of bacteria and it has been shown that NLRP3 is activated by the lung pathogenic microorganisms* K. pneumoniae*,* Listeria monocytogenes* [84, 85],* S. pneumoniae*,* S. aureus* [86],* C. pneumoniae* [87],* M. tuberculosis* [88],* L. pneumophila* [89], influenza virus [90, 91],* Porphyromonas gingivalis* [92],* Aspergillus fumigatus* [93], and* Aeromonas veronii* [94]. NLRP3 seems to be involved in the host defense against the enteric pathogens* Citrobacter rodentium* and* Clostridium difficile* in mice [62]; however, this response is far from being fully characterized.

Although NLRP1 was the first NLR described as a part of an inflammasome, its mechanism of activation is not well studied. It is abundantly expressed in lymphocytes, respiratory epithelial cells, and myeloid cells [95, 96]. The best-characterized activator of NLRP1 is the lethal toxin (LT) from* Bacillus anthracis* [97]; LT activates caspase-1 and induces rapid cell death via NLRP1 [81]. A recent work showed that NLPR1 inflammasome is activated by* T. gondii* in mice and rats infection models [98]. NLRP7 is only present in human peripheral blood mononuclear cells after LPS and IL-1*β* stimulation [99]. Despite its function in bacterial infections the experimental evidence indicates that NLRP7 is activated in macrophages by bacterial lipopeptides and* Mycoplasma* as well as* S. aureus* infection, leading to formation of an inflammasome [100].

NLRPs also negatively control the inflammatory response by lowering the NF-*κ*B activation and IFN*β* production [101, 102]. They regulate autophagy during group A streptococcal infection by interacting with the autophagy regulator Beclin-1 [103]. On the other hand, NLRP6 inhibits NF-*κ*B signaling downstream of TLRs in macrophages* in vitro* and mouse* in vivo* [104], which seems to be important to regulate the immune response against components of the gut microflora [105]. Also it has been described that ablation of* Nlrp6* confers resistance to* L. monocytogenes* and* Salmonella typhimurium* infections [104]. Although the lack of* Nlrp6* gene could be beneficial to control infection caused by these pathogens, it must be studied how its deficiency might affect the gut homeostasis. Another member of the NLRs family NLRP12 functions as a negative regulator of inflammation. It is expressed in myeloid cells and its expression is reduced by TNF*α* and TLR stimulation [106, 107]. However, a recent report has demonstrated that NLRP12 does not significantly contribute to the* in vivo* host innate immune response to LPS stimulation,* K. pneumoniae* infection, or* M. tuberculosis* [108].

Early experiments revealed that flagellin delivered into the cytosol is an important bacterial component for the activation of the NLRC4 inflammasome independently of TLR5 activation [109]. Besides NLRC4 regulates of host defense by activating caspase-1 and IL-1*β*/IL-18 secretion in macrophages infected with* Salmonella enterica* serovar Typhimurium,* L. pneumophila,* and* P. aeruginosa.* [110, 111].* S. flexneri*, a pathogen bacterium lacking flagellum, also induces the activation of the NLRC4 inflammasome through PrgJ, a protein that forms the basal body rod of the type three secretion system [112–114]. Furthermore, NLRC4 protects the gut from chemically induced acute colitis as well as the mortality caused by dissemination of* Salmonella* beyond the gut [115].

On the other hand, the absent in melanoma-2 (AIM2) protein is a member of the IFI20X/IFI16 (PYHIN) protein family that binds DNA from virus and bacterial pathogens. Upon DNA sensing, AIM2 triggers the assembly of the inflammasome, leading to caspase-1 activation, IL-1*β* maturation and pyroptotic cell death [116, 117]. Several studies have shown that AIM2 inflammasome is important in the recognition of DNA from pathogen bacteria, such as* Francisella tularensis* in macrophages [118],* Francisella novicida* in dendritic cells [119],* L. monocytogenes* in macrophages [84, 120–122],* Mycobacterium *sp. in macrophages [123, 124],* S. pneumoniae* in macrophages [125], and* P. gingivalis* in gingival epithelial cells [92]. Even AIM2 inflammasome is a critical molecular platform for regulating IL-1*β* release and survival during acute central nervous system (CNS)* S. aureus* infection [126].

## 6. Signaling Activated in Response to PAMPs

Initially, sensing of pathogenic bacteria by host activate signaling pathways that turn on mechanisms to kill the microorganism [2]. However, when the infection and inflammatory response have been resolved different mechanisms are launched to repair any tissue damage and return to the basal state [127]. This means that initiation, control, and termination of the inflammatory response and infection must be highly regulated. The inflammatory response is under the control of the NF-*κ*B, AP-1 or IRFs transcription factors, which driving the expression of genes that mediate several processes such as cell proliferation and release of antimicrobial molecules and cytokines that regulate the immune response [128]. In the following sections, the signaling mechanisms activated by TLRs, NLRs, and the collaborative action of both are discussed.

### 6.1. TLRs Signaling

**Figure 1:**
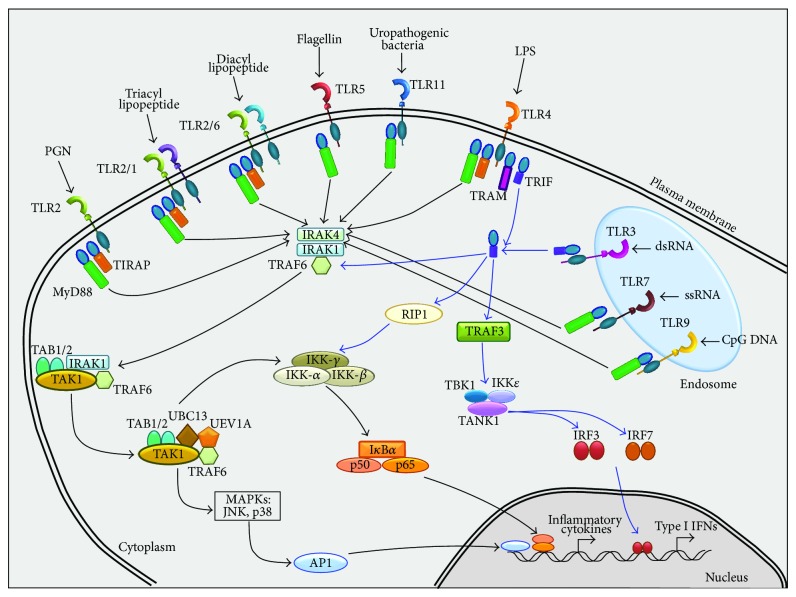
*TLRs signaling*. TLR signaling is activated in a MyD88-dependent (*black arrows*) and TRIF-dependent (*blue arrows*) manner. MyD88 signaling leads to NF-*κ*B activation, while TRIF signaling leads to both IRF3 and NF-*κ*B activation. In the MyD88-dependent ubiquitination of TRAF6 is important to activate MAPKs (JNK, p38 and ERK) or IKK complex to induce the translocation of NF-*κ*B to the nucleus. The TRIF-dependent signaling pathway can activate NF-*κ*B, IRF3, and IRF7.

TLR signaling pathways have been studied and reviewed extensively and it is known that they play a crucial role against pathogenic microbial infection through the induction of inflammatory cytokines and type I interferons (Type I IFNs) [11, 129, 130]. TLR signaling is activated in a myeloid differentiation primary response gene 88- (MyD88-) dependent and TIR-containing adaptor-inducing IFN-*β*- (TRIF-) dependent manner, with MyD88 signaling predominantly leading to the activation of NF-*κ*B, while TRIF signaling leading to both interferon regulatory factor 3 (IRF3) and, to a lesser extent, NF-*κ*B activation (Figure 1) [131, 132].

TLRs have an extracellular leucine-rich repeat (LRR) domain, a transmembrane domain and a cytoplasmic Toll/IL-1 receptor (TIR) domain. The LRR domain of TLRs is involved in the recognition of proteins (e.g. flagellin and porin from bacteria), carbohydrates (e.g. zymosan from fungi), lipids (LPS), lipid A, and lipoteichoic acid (LTA from bacteria), nucleic acids (CpG-containing DNA from bacteria and viruses and viral RNA), protein or peptide derivatives (lipoprotein and lipopeptides from various pathogens), lipid derivatives (LAM from mycobacteria), profilin from* T. gondii*, and diacyl-lipopeptides from mycoplasma [3, 133, 134]. On the other hand, the TIR domain of TLRs shows homology with the cytoplasmic region of the IL-1 receptor and interacts with TIR-domain-containing adaptors such as MyD88, TIR-containing adaptor protein (TIRAP), TRIF and TRIF-related adaptor molecule (TRAM) [135]. In the MyD88 signaling pathway, stimulation of TLRs triggers its association with MyD88, which in turn recruits IL-1R-associated kinase 4 (IRAK4), allowing the assembly of IRAK1. IRAK4 then induces the phosphorylation of IRAK1, which in turn interacts with tumor-necrosis-factor receptor-associated factor 6 (TRAF6). Phosphorylated IRAK1 and TRAF6 then dissociate from the receptor and form a complex with transforming-growth factor-*β*-activated kinase 1 (TAK1), TAK1-binding protein 1 (TAB1), and TAB2 at the plasma membrane, promoting the phosphorylation of TAB2 and TAK1. IRAK1 is degraded at the plasma membrane and the remaining complex, consisting of TRAF6, TAK1, TAB1, and TAB2, associates with the ubiquitin ligases, ubiquitin-conjugating enzyme 13 (UBC13) and ubiquitin-conjugating enzyme E2 variant 1 (UEV1A) in the cytosol. Ubiquitination of TRAF6 induces the activation and phosphorylation of mitogen-activated protein kinases (MAPKs), Jun N-terminal kinase (JNK), p38, extracellular signal-regulated kinase (ERK), and the inhibitor of nuclear factor-*κ*B- (I*κ*B-) kinase (IKK) complex, which consists of IKK-*α*, IKK-*β*, and IKK-*γ* (also known as IKK1, IKK2, and NEMO, resp.) [131, 132, 135]. The IKK complex then phosphorylates the inhibitor of NF-*κ*B (I*κ*B), which leads to its ubiquitination and subsequent degradation by the proteasome 26S, allowing the translocation of NF-*κ*B to the nucleus and expression of inflammatory cytokines, chemokines, costimulatory molecules, and other effectors necessary to build up the host cell “*weapons*” against the invading pathogen [129, 136, 137]. The variety of genes induced to express by TLRs may be due to the existence of several adaptors that possess TIR domains. Except for TLR3 all TLRs recruit MyD88 and only TLR1, TLR2, TLR4, and TLR6 recruit the additional adaptor TIR-domain-containing adaptor protein (TIRAP, also known as MyD88-adaptor-like protein, MAL) that functions as a bridge between the TIR domain and MyD88 [1, 131, 135, 138].

Apart from the activation of NF-*κ*B and MAP kinases, the TRIF-dependent signaling pathway also induces the activation of IFN*β*. TRIF contains a Rip homotypic interaction motif (RHIM) in its C-terminal region that mediates the interaction with members of the receptor-interacting protein (RIP) family. It was observed that TRIF activates NF-*κ*B either by direct interaction with TRAF6 or through RIP-1. Both TRIF/RIP-1 and TRIF/TRAF6 pathways converge at the IKK complex to achieve maximum activation of NF-*κ*B-dependent gene expression. Expression of the IFN*β* gene is controlled by cooperative activation of NF-*κ*B, ATF2/c-Jun, IRF3, and IRF7. Activation of TRAF3 by TRIF is important to generate a link between TRIF and TANK-binding kinase 1 (TBK1, also known as NF-*κ*B activating kinase, NAK), and this in turn activates TBK1 and IKK*ε*. TBK1 and IKK*ε* then activate these two molecules that are responsible for the activation of TRAF family member-associated NF-*κ*B activator (TANK), which phosphorylates IRF3 and IRF7. Phosphorylated IRF3 and IRF7 form homodimers and move to the nucleus where it binds to IFN-stimulated response elements (ISRE), resulting in the production of type I IFNs and IFN stimulatory genes (ISGs) [4, 139–142]. Although IRF7 is considered as the master regulator of IFN-*α* response [143], IRF5 also seems to function downstream of TLR7 or TLR9, and perhaps TLR8 signaling, although its expression is mainly restricted to B cells, macrophages, monocytes and dendritic cells where can induce de INF-*α* production [144, 145]. Moreover, IRF1 has been identified as a downstream signaling element of TLR7 in dendritic cell infected with* Candida albicans* [146, 147].

### 6.2. NLRs Signaling

**Figure 2:**
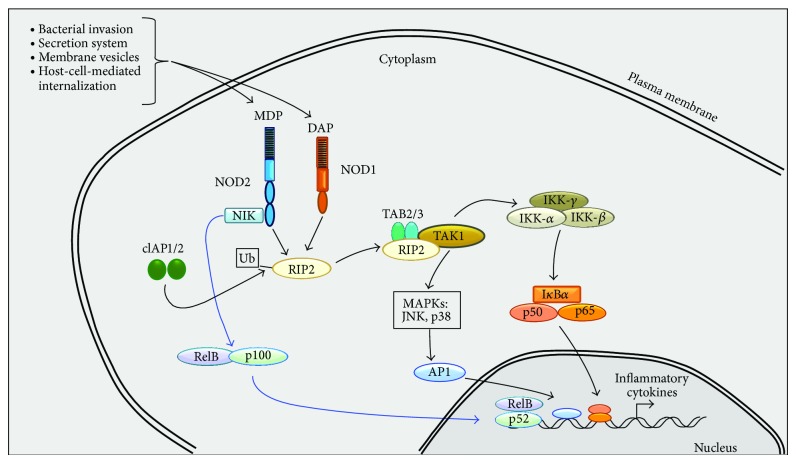
*NOD1 and NOD2 signaling.* Bacteria or their components reach the cytosol by several mechanisms. The interaction between NOD2 and NIK (*blue arrows*) activates the noncanonical NF-*κ*B pathway (p100/p52-dependent). While binding of PGN to LRR domain of NLRs leads to recruitment of RIP-2 through CARD-CARD interaction (*black arrows*). Ubiquitination of RIP-2 favors, on the one hand, the activation of the MAPKs p38/JNK, and on the other hand activates IKK complex, which induces the activation of NF-*κ*B.

As stated above, the NLRs NOD1 and NOD2 regulate proinflammatory cytokine expression induced by intracellular bacterial ligands. NOD1 recognizes mainly Gram-positive PGN fragments containing the* N-*acetylglucosamine-*N*-acetylmuramic acid tripeptide motif with diaminopimelic acid (DAP). NOD2 detects muramyl dipeptide (*N*-acetylmuramic acid-L-alanyl-D-isoglutamine), which is a motif common of PGN from both Gram-negative and Gram-positive bacteria [148–152]. In general, NLRs possess a C-terminal LRR domain, often involved in ligand recognition, a central NOD, and a variable N-terminal effector domain that is used to classify NLRs [153]. Once bacteria or their components reach the host cytosol by phagocytosis, invasion, membrane vesicles, or secretion systems, the interaction of NLRs with PGN takes place [60, 154], although whether it is a direct or indirect contact is still unclear. However, it has been well documented that the inflammatory response initiated by NOD1 and NOD2 induces the expression of proinflammatory cytokines, chemokines, and antimicrobial peptides by activating NF-*κ*B and AP-1 (Figure 2) [153, 155–157]. A direct interaction between NOD2 and NF-*κ*B-inducing kinase (NIK) that triggers the p100/p52-dependent induction of the noncanonical NF-*κ*B pathway was demonstrated by Pan et al. [158]. Although the NLR signaling pathway is far from being fully characterized the general model shows that sensing of PGN leads to transient recruitment of RIP-2 through CARD-CARD interaction [151, 159–161]. The RIP-2 recruitment leads to IKK complex activation and the subsequent NF-*κ*B activation through phosphorylation and ubiquitination of I*κ*B*α*, inducing proinflammatory cytokines production [51, 162, 163]. Moreover, recruitment of RIP-2 by NOD1 also activates JNK [164], and NOD1/2 seems to participate in the activation of the type I IFN pathway [165]. Cellular inhibitor of apoptosis protein 1 and 2 (cIAP1 and cIAP2) are E3 ubiquitin ligases important for ubiquitination of RIP-2 and for signaling downstream of both NOD1 and NOD2. Polyubiquitinated RIP-2 favors the recruitment and activation of the TAK1-TAB2/3 complex. TAK1 in turn phosphorylates and activates the MAPKs p38/JNK and NF-*κ*B pathways, leading to cytokine, chemokine, and antimicrobial peptide production [154, 160, 166, 167].

## 7. Combined Response of TLRs and NLRs Signaling

As it was explained earlier TLRs and NLRs regulate the cytokine and chemokine expression in response to bacterial ligands through their respective signaling pathways [72, 168, 169]. It is likely that TLRs and NLRs act in a collaborative/synergistic, complementary, or compensable manner, with the aim to increase the sensitivity to detect and efficiently eliminate pathogenic bacteria. A number of reports that analyze the interaction between TLRs and NLRs have been published (Table 2). In human monocytic THP-1 cells a marked synergistic secretion of IL-8 was induced by synthetic agonists of NOD1/2 and TLR2/4/9. This enhanced IL-8 mRNA expression and NF-*κ*B activation was suppressed in NOD1 and NOD2 genes silenced monocytes [170]. This synergism between NOD1/2 and TLRs was also observed in the production of antimicrobial peptides PGN recognition proteins (PGRPs) and *β*-defensin 2 in human oral epithelial cells via NF-*κ*B [171]. Interestingly, costimulation of NOD1/2 and TLRs did not have any effect on IL-8 production, which suggests a cell-type specific inflammatory response. Likewise, in human monocytes and dendritic cells the NOD1/2 ligands, DAP and MDP, respectively, exert a synergetic activity with LPS in the expression of proinflammatory cytokines TNF-*α*, IL-1*β*, IL-6, and IL-8 [172]. The synergistic effect of MDP and TLR2/3/4 ligands on IL-6 and IL-12p40 expression was also observed in wild type and NOD2^−/−^ macrophages, via NF-*κ*B, p38 and ERK signaling pathways [173]. Additionally, treatment of human dendritic cell (DC) with MDP and the NOD1 agonist FK565 along with TLR3/4/9 agonists synergistically induced IL-12p70 and INF-*γ* production [174]. Another NOD1 ligand M-TriDAP markedly increased the response induced by LPS for multiple cytokines such as IL-1*α*, IL-1*β*, IL-4, IL-6, GM-CSF, IL-10, and TNF-*α*. In the same study carried out by van Heel et al. [175] a strong synergistic increase in IL-1*β* production was observed for TLR1/2, TLR2/6, TLR4, TLR5, and TLR7/8 ligands (Pam_3_CysLys_4_; MALP2; LPS; flagellin from* S. typhimurium* and R-848, resp.) combined with M-TriDAP. Assays performed with homozygotic macrophages for the 3020*insC* mutation and/or TLR2^−/−^ mice demonstrated that the NOD2 ligand MDP has a synergistic effect on the induction of TNF-*α*, IL-1*β*, and IL-10 upon costimulation with specific TLR2 agonists Pam_3_Cys and MALP2 [176].

In a more recent work, it was shown that NOD1 and TLR2 cooperate to enhance human CD8 T cells proliferation and expansion, and this cooperating action caused an enhanced secretion of IL-2, IFN-*γ*, and TNF-*α* that was related to increased activation of NF-*κ*B, JNK, and p38 signaling pathways [177]. Simultaneous stimulation of monocytes-derived DC with NOD1 and NOD2 ligands combined with TLR7/8 or TLR4 agonists results in highly increased production of IL-1*β* and IL-23 and expression of the inhibitor suppressor of cytokine signaling 2 (SOCS2), where the NOD1/TLR agonists combination was more relevant for the synergistic activity observed [178]. Altogether, these results clarify the existence of a cooperative action of TLRs and NLRs, which become relevant in the context of infection by bacteria that can be recognized by extra- and intracytosolic receptors. Ferwerda et al. [179] demonstrate that TLR2 and NOD2 are two nonredundant receptors that sense* M. tuberculosis*. They found a synergistic action between these two receptors for cytokine expression that was lost in cells from individuals homozygous for NOD2 3020*insC* mutation or macrophages harvested from TLR2^−/−^ mice (Table 3). The same authors also found that macrophages from TLR2 and TLR4 knockout mice and NOD2 mutant had a decreased production of TNF-*α*, IL-1*β*, IL-6, and IL-10, which suggest that collaborative activity of these PRRs is important to balance the amount of pro- and anti-inflammatory response in* M. paratuberculosis *infection [179]. Evidence on the collaborative activity between TLR2 and NOD2 was obtained by measuring IL-10 secreted from macrophages challenged with* S. pneumoniae* cell-wall (PnCW) fragments [180]. Interestingly, in this IL-10 production participated the protein adaptors RIP-2 and MyD88, reflecting that both canonical signaling pathways were involved. Infection of mice mesothelial cells (MC) with* L. monocytogenes* caused an increased production of CXCL1 and CCL2 chemokines, which was notably decreased in MC deficient in NOD1 and RICK [160].

*C. pneumoniae*, which is a common pathogen that causes pneumonia in humans can be recognized by TLR2 and TLR4 [181], as well as by NOD1/2 [70]. Sensing of* C. pneumoniae* by these receptors induced a reduction in the expression of IL-6, IL-12p40, and IFN-*γ* in RIP-2^−/−^ mice at day 3 after infection compared with wild-type mice. However, at days 5 and 14 after infection, the production of these cytokines was significantly increased in wild-type mice, indicating an initial impaired and delayed kinetics of cytokine production in* C. pneumonia*-infected RIP-2^−/−^ mice. Thus, the collaborative activity of both NLRs and TLRs is fundamental for efficient pathogen bacterial clearance. This idea has been supported by later experiments in which NLRC4 was necessary to eliminate* L. pneumophila*, while TLR5 was necessary to recruit neutrophils [182]. Furthermore, it has been demonstrated that activation of RIP2-, MyD88-, and Naip5/NLRC4-dependent signaling pathways triggers a coordinated and synergistic response that protects the host against lethal infection by* L. pneumophila* [183]. However, contrasting results were observed in* P. aeruginosa* macrophage infection, since NLRC4 and caspase-1 activation attenuated the autophagy activated by TLR5 and reduced the type I interferon production [184], besides NLRC4 dampens a beneficial IL-17-mediated antimicrobial host response through IL-18 secretion [185].

On the other hand, in DCs infected with* Helicobacter pylori*, the cooperative interaction between TLR2 and NOD2 showed to be important for IL-1*β* production and NLRP3 activation. This cooperative interaction in* H. pylori* infection was confirmed in IL-1*β*- and IL-1 receptor-deficient mice in which the clearance of bacteria from the stomach was impaired compared with wild-type mice [186]. Costimulation of BALB/c mice with NOD1 and TLR5 ligands showed to be important for efficient* S. enterica* clearance and improved mice survival. This effect was accompanied with an increase in IL-5, IL-6, IL-13, IL-21, IL-22, TNF-*α*, and *β*-defensin 3 in small intestine [187]. More evidence of NOD2 and TLR2 cooperative action was obtained in DC stimulated with PGN from* S. aureus* [188]. Analysis of IL-6 and IL-1*β* production, revealed an additive effect of both receptors in keratinocytes from murine oral epithelium, since in TLR2- or NOD2-deficient keratinocytes the cytokine release was decreased by approximately 50% compared to wild-type cells [189]. Infection of mouse macrophages with* Vibrio vulnificus* and* Vibrio cholerae* revealed the activation of caspase-1 via the NLRP3 inflammasome [190]. In this work, experiments made with mice doubly deficient in MyD88 and TRIF (*Myd* ^*−/−*^ *Trif* ^*−/−*^), demonstrated that NLRP3 activation required NF-*κ*B-dependent TLR stimulation. Altogether, these results indicate that several sensors are necessary to fight against pathogenic bacteria. Moreover, the specific combination of PRRs seems to be coordinated according to the bacteria or PAMPs involved, which subsequently affect the host response by driving collaborative/synergistic activity.

## 8. Final Considerations

Most studies have focused on the characterization of the inflammatory response triggered by several virulence factors alone. However, it is important to take into account that in physiological conditions the participation of several PRRs that respond to different PAMPs could be more effective for the host to combat infections. Regarding this issue, the experimental evidence accumulated so far has pointed out that the host response against pathogenic bacteria may be the sum of several pathways induced by the recognition of different PAMPs by different PRRs, which in turn trigger and shape the subsequent innate and adaptive immune responses. Although synergistic activity among NLRs and TLRs has been demonstrated, the subjacent mechanisms are not clear. Both receptors are able to activate NF-*κ*B, MAPKs, AP-1, or IRFs signaling pathways; however, the molecules that are involved in the synergistic activation of these pathways have not been identified. Then, for a better understanding of the molecular mechanisms by which NLRs and TLRs collaborate, this synergistic activity will have to be further analyzed. Since TRLs and NLRs play a fundamental role in the eradication of invading pathogen microorganisms through the induction of inflammatory and antimicrobial peptides, this collaborative activity could be exploited to modulate or improve the host response against pathogenic bacteria that causes an exacerbated inflammatory response.

Finally, there is a growing interest in targeting these PRRs for the treatment of sepsis, but also to fight against inflammatory diseases such as cancer, rheumatoid arthritis, inflammatory bowel disease, and systemic lupus erythematosus [191, 192]. Several approaches, like ligand mimetics to activate PRRs and antibodies or molecules to inhibit them, have been used to identify therapeutics targets [193]. However, something that should be taken into account in the development of these strategies is the collaborative activity among different PRRs.

## Figures and Tables

**Table 1:** Expression and localization of TLRs and NLRs in cellular types.

Receptor	Cellular type	Localization	Reference
TLR1	Monocytes, mature macrophages, mast cell, and dendritic cells	Cell surface	[191]
TLR2	Monocytes, mature macrophages, and mast cell	Cell surface	[191]
TLR3	Dendritic cells	Endosomes	[191]
TLR4	Predominately in monocytes, mature macrophages, dendritic cells, mast cells, and intestinal epithelial cells	Cell surface	[191]
TLR5	Predominately in intestinal epithelial cells, monocytes, macrophages, and dendritic cells	Cell surface	[191]
TLR6	Monocytes, mature macrophages, and mast cell	Cell surface	[191]
TLR7	Monocytes, macrophages, and plasmacytoid dendritic cells	Endosomes	[191]
TLR8	Monocytes, macrophages, and mast cells	Endosomes	[191]
TLR9	Monocytes, macrophages, and plasmacytoid dendritic cells	Endosomes	[191]
TLR10	Macrophages, trophoblasts, and intestinal epithelial cells in response to *L. monocytogenes *	Cell surface, but can colocalize with TLR2 in phagosome	[23–25]
TLR11	Macrophages, dendritic cells, and human embryonic kidney cells	Cell surface and endoplasmic reticulum	[26, 27]
TLR12	Macrophages and dendritic cells	Colocalizes with TLR11 in endoplasmic reticulum	[26, 27]
NOD1	Macrophages, human mononuclear cells, intestinal epithelial cells, and dendritic cells	Intracellularly	[50–52]
NOD2	Macrophages, neutrophils, dendritic cells, Paneth cells, human airway smooth muscle cells, and epithelial and endothelial cells	Intracellularly	[50–52]
NLRC4	Macrophages and gut epithelial cells	Intracellularly	[109, 111, 114]
NLRP1	Lymphocytes, respiratory epithelial cells, and myeloid cells	Intracellularly	[95, 96]
NLRP3	Myeloid cells and human bronchial epithelial cells	Intracellularly	[82]

**Table 2:** Analysis of the collaborative action of TLRs and NRLs using synthetic agonists.

Cell type	Chemical agonist used	Receptors involved	Cellular response
Human monocytic leukemia THP-1	iE-DAP and MDP Pam3CSSNA, LA-15-PP, CpG DNA	NOD1 and NOD2 TLR2, 4 and 9	NF-*κ*B activation, IL-8 mRNA expression and secretion of IL-8

Human oral epithelial HSC-2	FK156 and MDP FSL-1, Poly I:C, LA-15-PP, Poly U and CpG DNA	NOD1 and NOD2 TLR2, 3, 4, 7 and 9	NF-*κ*B activation, and production of PGRPs and *β*-defensin 2

Human embryonic kidney HEK293T and human monocytes	MtriDAP and MDP or MtriLYS Pam_3_CSK_4_ and LPS preparations from *Escherichia coli* O111:B4	NOD1 and NOD2 TLR2	Release of TNF-*α*, IL-1*β*, IL-6, and IL-8

Bone marrow-derived mice macrophages	MDP Pam_3_CSK_4_, Poly I:C and LPS	NOD2 TLR2, 3 and 4	Activation of NF-*κ*B, p38 and ERK. Production of IL-6 and IL-12p40

Human dendritic cells	FK565 and MDP Poly I:C, LA-15-PP, and CpG DNA	NOD1 and NOD2 TLR3, 4 and 9	Production of IL-12p70, IL-15, and IFN-*γ*

Human blood mononuclear cells	MtriDAP LPS, MALP2, Pam_3_CysLys_4_, Flagellin from *Salmonella typhimurium *and R-848	NOD1 TLR4, 2/6, 1/2, 5 and 7/8	Secretion of TNF-*α*, IL-1*α*, IL-1*β*, IL-4, IL-6, IL-10, and GM-CSF

Human homozygotic macrophages (3020*insC* mutation)	MDP Pam_3_Cys and MALP2	NOD2 TLR2	Production of TNF-*α*, IL-1*β*, and IL-10

Murine and human CD8 T cells	iE-DAP Pam_3_CSK_4_	NOD1 TLR2	Activation of NF-*κ*B, p38 and JNK Secretion of TNF-*α*, IL-2, and, IFN-*γ* Induction of cellular proliferation and expansion

Monocyte derived-dendritic cells	iE-DAP and MDP LPS and R848	NOD1 and NOD2 TLR4 and 7/8	Secretion of IL-1*β* and IL-23; expression of SOCS2

**Table 3:** Analysis of the collaborative action of TLRs and NRLs in response to pathogenic bacteria.

Organism/Cell type	Pathogen bacteria	Receptors involved	Cellular response evaluated
Human homozygotic macrophages (3020*insC* mutation) and macrophages from TLR2^−/−^ or WT mice	*Mycobacterium tuberculosis *	NOD2 and TLR2	Production of TNF-*α*, IL-1*β* and IL-6

Human homozygotic macrophages (3020*insC* mutation) and macrophages from TLR2^−/−^, TLR4^−/−^, MyD88^−/−^ or WT mice	*Mycobacterium paratuberculosis *	NOD2, TLR2 and TLR4	Production of TNF-*α*, IL-1*β*, IL-6 and IL-10

Bone marrow-derived macrophages from TLR2^−/−^, NOD2^−/−^ or WT mice	*Streptococcus pneumoniae* cell wall (PnCW)	NOD2 and TLR2	Production of IL-10

Mesothelial cells from RICK^−/−^, NOD1^−/−^ or WT mice	*Listeria monocytogenes *	NOD1 and TLR2	Production of CXCL1 and CCL2

NOD1^−/−^, NOD2^−/−^, RIP2^−/−^ or WT mice	*Chlamydophila pneumoniae *	NOD1, NOD2, TLR2 and TLR4	Expression of iNOS, production of NO, IL-6, IL12p40 and IFN-*γ*, and bacterial clearance

NLRC4^−/−^/TLR5^−/−^ or WT mice	*Legionella pneumophila *	NLRC4 and TLR5	Bacterial clearance, recruitment of neutrophils

MyD88^−/−^, RIP2^−/−^, WT mice or with nonfunctional *Naip5* gene	*Legionella pneumophila *	NLRC4, TLR2, TLR5 and TLR9	Replication and dissemination of bacteria, rates of mortality and production of IL-6

NOD1^−/−^, NOD2^−/−^, NLRP3^−/−^, NLRC4^−/−^, Casp1^−/−^, Asc^−/−^, TLR2^−/−^ or WT mice	*Helicobacter pylori *	NOD2, TLR2 and NLRP3	Production of IL-1*β* and NLRP3 activation

BALB/c mice and human monocyitic leukemia THP-1	*Salmonella enterica* serovar Typhimurium	NOD1 and TLR5	Bacterial clearance and production of IL-5, IL-6, IL-13, IL-21, IL-22, TNF-*α* and *β*-defensin 3

Dendritic cell Keratinocytes from oral epithelium	*Staphylococcus aureus *	NOD2 and TLR2	Production of IL-6 and IL-1*β*

Mice double deficient in *Myd* ^−/−^ *Trif* ^−/−^	*Vibrio vulnificus *and *Vibrio cholerae *	TLRs and NLRP3	Activation of NF-*κ*B

Mice and primary monocytes	*Pseudomonas aeruginosa *	TLR5 and NLRC4	Autophagy and production of IL-17, IL-18 and Type I IFN
